# Comparative immunogenicity of an mRNA/LNP and a DNA vaccine targeting HIV *gag* conserved elements in macaques

**DOI:** 10.3389/fimmu.2022.945706

**Published:** 2022-07-22

**Authors:** Antonio Valentin, Cristina Bergamaschi, Margherita Rosati, Matthew Angel, Robert Burns, Mahesh Agarwal, Janina Gergen, Benjamin Petsch, Lidia Oostvogels, Edde Loeliger, Kara W. Chew, Steven G. Deeks, James I. Mullins, George N. Pavlakis, Barbara K. Felber

**Affiliations:** ^1^ Human Retrovirus Section, Vaccine Branch, Center for Cancer Research, National Cancer Institute at Frederick, Frederick, MD, United States; ^2^ Human Retrovirus Pathogenesis Section, Vaccine Branch, Center for Cancer Research, National Cancer Institute at Frederick, Frederick, MD, United States; ^3^ Vaccine Branch, Center for Cancer Research, National Cncer Institute, Bethesda, MD, United States; ^4^ Center for Cancer Research Collaborative Bioinformatics Resource, Leidos Biomedical Research, Inc., Frederick National Laboratory for Cancer Research, Frederick, MD, United States; ^5^ CureVac AG, Tübingen, Germany; ^6^ Department of Medicine, David Geffen School of Medicine at University of California, Los Angeles, Los Angeles, CA, United States; ^7^ Division of HIV, Infectious Diseases and Global Medicine, University of California, San Francisco, CA, United States; ^8^ Department of Microbiology, University of Washington, Seattle, WA, United States; ^9^ Department of Medicine, University of Washington, Seattle, WA, United States; ^10^ Department of Global Health, University of Washington, Seattle, WA, United States

**Keywords:** mRNA/LNP, therapeutic immunization, HIV, T cell response, antibody, *gag*, conserved sequences, immune focusing

## Abstract

Immunogenicity of HIV-1 mRNA vaccine regimens was analyzed in a non-human primate animal model. Rhesus macaques immunized with mRNA in lipid nanoparticle (mRNA/LNP) formulation expressing HIV-1 Gag and Gag conserved regions (CE) as immunogens developed robust, durable antibody responses but low adaptive T-cell responses. Augmentation of the dose resulted in modest increases in vaccine-induced cellular immunity, with no difference in humoral responses. The *gag* mRNA/lipid nanoparticle (LNP) vaccine provided suboptimal priming of T cell responses for a heterologous DNA booster vaccination regimen. In contrast, a single immunization with *gag* mRNA/LNP efficiently boosted both humoral and cellular responses in macaques previously primed by a *gag* DNA-based vaccine. These anamnestic cellular responses were mediated by activated CD8^+^ T cells with a phenotype of differentiated T-bet^+^ cytotoxic memory T lymphocytes. The heterologous prime/boost regimens combining DNA and mRNA/LNP vaccine modalities maximized vaccine-induced cellular and humoral immune responses. Analysis of cytokine responses revealed a transient systemic signature characterized by the release of type I interferon, IL-15 and IFN-related chemokines. The pro-inflammatory status induced by the mRNA/LNP vaccine was also characterized by IL-23 and IL-6, concomitant with the release of IL-17 family of cytokines. Overall, the strong boost of cellular and humoral immunity induced by the mRNA/LNP vaccine suggests that it could be useful as a prophylactic vaccine in heterologous prime/boost modality and in immune therapeutic interventions against HIV infection or other chronic human diseases.

## Introduction

The introduction of highly efficient antiretroviral drugs (ART) for the treatment of HIV infection dramatically improved the disease prognosis and extended the life expectancy of infected individuals [reviewed in (1–6)]. Nevertheless, ART fails to eradicate infected cells, and upon ART discontinuation, viral rebound occurs within 2-4 weeks. Therefore, life-long continuous ART is required to prevent disease progression. To eliminate the burden of chronic drug consumption and associated long-term toxicities, immune therapeutic strategies aiming to eliminate the long-term reservoir of HIV-infected cells or achieve a functional cure are being explored.

Therapeutic vaccination has a potential role either as a component of a strategy to eliminate cells latently infected with HIV-1 (reduction of latent reservoir), or as a functional cure to achieve permanent host control of HIV-1 infection to undetectable levels off ART without complete eradication of the latent reservoir (7–9). Due to control of virus replication under ART, only very low or no virus-specific T cell responses are present in the circulation. An effective therapeutic HIV-1 vaccine should induce potent cytotoxic T cell responses which could contribute to control of viremia and thereby reduce the pool of infected cells. CD8^+^ T cell immune responses induced upon therapeutic vaccination during ART can contribute to control viral replication upon treatment interruption [reviewed in (6–8, 10–13)].

We and others have introduced the concept of directing T cell responses towards conserved regions in the HIV proteome (13–24). Our initial approach (15, 16), using the DNA-based vaccine platform, targeted conserved elements (CE) within HIV-1 p24^Gag^ (14, 25, 26). CE were selected following stringent criteria: (i) more than 98% conservation among the known HIV-1 sequences, (ii) prevalent recognition by long-term non-progressor HIV-infected individuals, and (iii) encoding of conserved epitopes with very broad HLA coverage at the population level. We showed that mutations in Gag CE are much more likely to disable virus replication in cell culture than mutations outside of CE (26–28). We demonstrated that vaccination with plasmid DNA encoding these CE epitopes is immunogenic in murine and NHP models (15, 16, 29). We reported novel CE vaccination regimens that modified the hierarchy of T cell epitope recognition otherwise imposed by the dominant variable regions within the full-length viral proteins (16, 30). These optimized DNA vaccine regimens, aiming to induce an adaptive response that makes virus escape difficult, broadened epitope recognition and improved the functionality of the vaccine-induced T cell responses, eliciting cytotoxic T cells targeting conserved epitopes in immunized rhesus macaques.

Using the SIV/macaque model, we have also shown that DNA vaccines expressing homologous epitopes present in SIV p27^Gag^ were very immunogenic (31). The T cells targeting these conserved epitopes were activated upon SIV-infection which demonstrated that the CE-specific T cells recognize infected cells *in vivo*. Thus, the use of immunogens encoding CE epitopes may be a promising therapeutic strategy for the management of HIV-1 infected individuals. The concept of CE vaccination has been translated into several clinical trials, including one prophylactic trial in HIV-naïve human volunteers (HVTN 119; NCT03181789) and two therapeutic trials in HIV-positive individuals on ART (ACTG A5369 [NCT03560258] and NCT04357821).

Nucleic acid-based vaccines have several significant advantages over other vaccine platforms, including streamlined and predictable scale-up production, and flexibility to enable rapid vaccine design. These features are critical in global outbreak situations and against emerging infectious diseases (locally or globally) (32). In addition, nucleic acid-based vaccines are not limited in the number of vaccinations because, in contrast with other modalities, especially viral vector-based vaccines, they do not induce immune responses targeting any vaccine component other than the intended immunogen [reviewed in (33–38)]. A putative hurdle with DNA vaccines is the delivery, that is performed by intramuscular/intradermal injection, and requires nuclear entry for immunogen expression, a process that is augmented by i.e., *in vivo* electroporation and the rare chance of integration into the genome [reviewed in (39, 40)].

In contrast, mRNA-based vaccines only require entry into the cytoplasm for translation, and this is achieved by simple needle/syringe injection. However, mRNA needs to be formulated within nanoparticles to avoid degradation and facilitate cellular uptake. LNP formulated mRNA vaccines may have an adjuvant effect by stimulating several innate immune responses and induce cytokine release shortly after immunization, which could influence the development of an efficient adaptive immune response (41). Although both the BNT162b2 (Pfizer-BioNTech) and mRNA-1273 (Moderna) demonstrated powerful vaccine efficacy against COVID-19, they are also associated with an excess risk of myocarditis, the level of adverse reactions with additional vaccine doses needs to be understood (42–44).

Among the nucleic acid-based vaccines, the DNA platform elicits long-lasting adaptive responses with both CD4^+^ and cytotoxic CD8^+^ T cell responses in macaques and humans (30, 45–52). mRNA vaccines are very efficient in inducing humoral immunity and mainly CD4^+^ T helper responses against several antigens (32, 35, 36, 53–56). The successful development and practical application of the mRNA technology have been showcased with the recent approval and distribution of several COVID-19 mRNA vaccines, demonstrating induction of potent anti-Spike Ab and low levels of CD4^+^ T helper and CD8^+^ T cell responses in humans (55, 57–64).

No comparative studies of the two nucleic acid vaccine modalities using the same immunogens have been reported so far. In this study, we tested the HIV-1 CE vaccine concept using an mRNA/LNP vaccine platform developed by CureVac to explore its immunological potential as T cell vaccine in Indian rhesus macaques. This technology comprised of chemically non-modified nucleoside synthetic mRNAs has been tested in pre-clinical and clinical trials (35, 65–68). The immunological outcome of this study was also compared to similar DNA based vaccine regimens. In addition, we evaluated combinations of DNA and RNA vaccine technologies in different prime-boost immunization studies, identifying approaches to further increase cellular immunity with promising immunological advantages.

## Materials and methods

### Animals and vaccines

**Figure 1:**
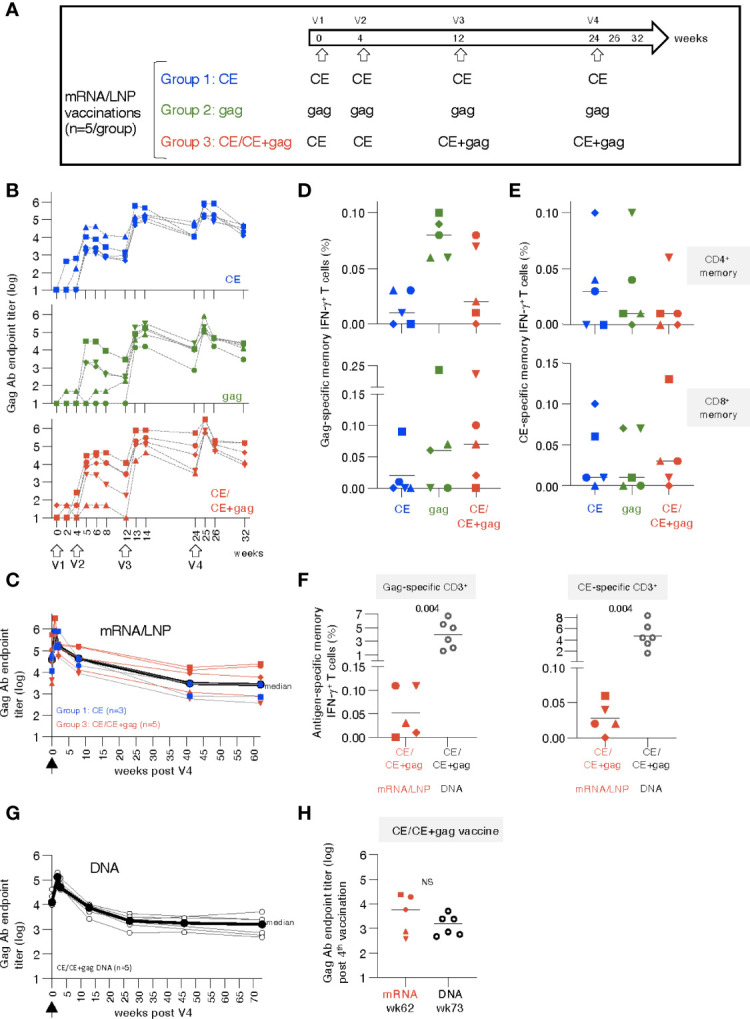
mRNA/LNP vaccination of naïve rhesus macaques induces robust antibody and low T cell responses. **(A)** Schematic representation of the HIV-1 mRNA/LNP vaccination regimens of the three groups receiving four vaccinations (V1 to V4) with the indicated gag immunogens. **(B)** Plots showing the vaccine-induced Gag Ab measured over time as reciprocal endpoint titers (log). **(C)** Durability of CE and CE+gag mRNA/LNP vaccine induced Gag antibodies. Gag Ab titers were plotted from eight vaccinated macaques [group 1, n=3 (CE mRNA/LNP), blue symbols; group 3, n=5, red symbols; (CE/CE+Gag mRNA)] described in panel B over 62 weeks post the last vaccination (V4). Black symbols denote median antibody titers. **(D, E)** Antigen-specific T cell responses by flow cytometry measured two weeks after the 4^th^ vaccination. **(D)** Gag-specific and **(E)** CE-specific memory (CD3^+^CD95^+^IFN-γ^+^) T cell responses were measured 2 weeks after the 4^th^ vaccination. **(F)** Comparison of memory T cell responses in macaques immunized with the mRNA/LNP regimen (group 3) and the homologous DNA vaccine regimen from historical samples (30) at two weeks post vaccination 4. The data were obtained in the same flow cytometer (BD Fortessa) using the same antibody panel and the same gating strategy in these two groups of samples and included an internal positive control. This approach excluded any variability associated with instrument and/or reagent performance. P values are from unpaired t test (Mann-Whitney). **(G)** The CE/CE+gag DNA vaccine (dose: 2 mg prime, 2 + 2 mg boost) contained IL-12 DNA as vaccine adjuvant and was administered by IM injection followed by electroporation using the same schedule for the matching the mRNA/LNP. Plot showing Gag Ab responses after the 4^th^ vaccination. The last time points of blood collection were weeks 70 and 76, respectively, for 3 animals each and these time points were combined plotted as week 73. **(H)** Comparison of Gag antibody titers (log) in macaques receiving CE/CE+Gag vaccine as mRNA/LNP (wk 62) and DNA (wk 73) vaccine post the 4^th^ vaccination, respectively.

**Figure 2:**
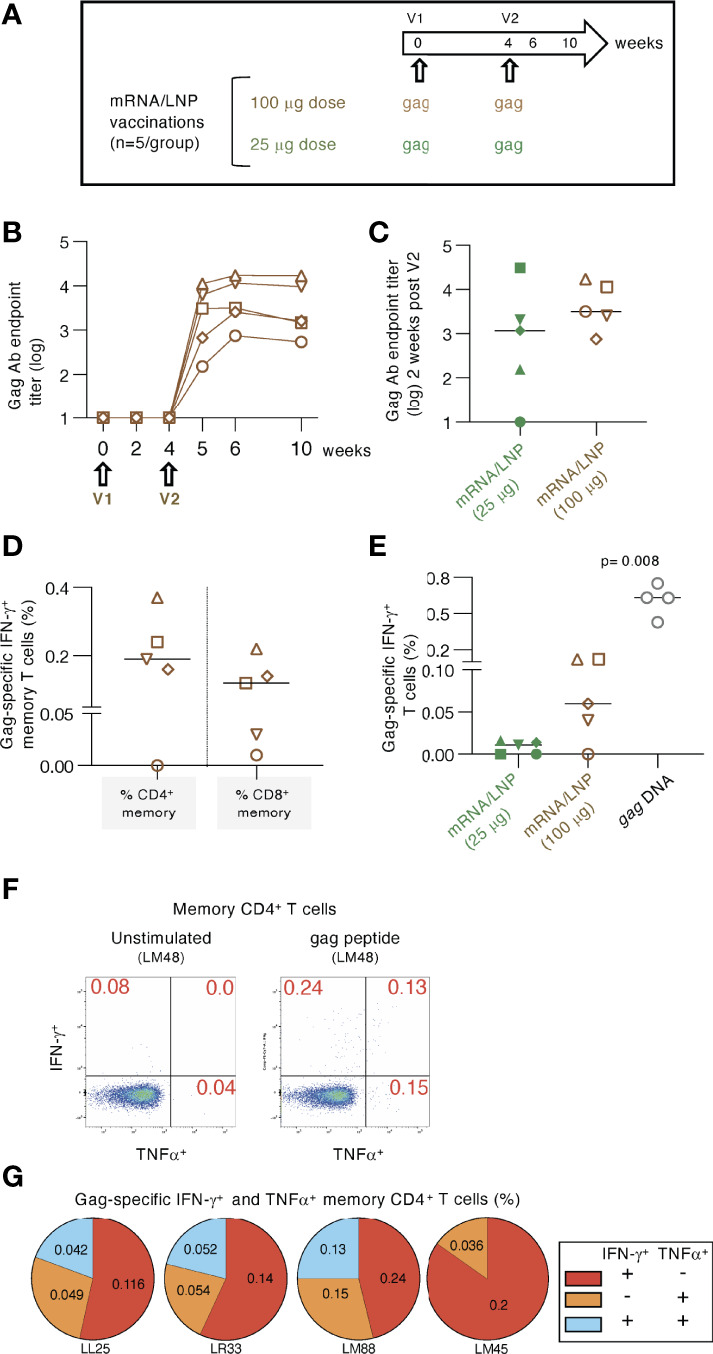
High dose mRNA/LNP vaccination increased cellular but not humoral responses. **(A)** Schematic representation of the high-dose (100 μg) *gag* mRNA/LNP vaccination regimen administered in two vaccinations (V1, V2; brown symbols). The data were compared to the low dose (25 μg) regimen described in **Figure 1** (group 2; green symbols). **(B)** Vaccine-induced Gag Ab titers were plotted over time as reciprocal endpoint titers (log) from macaques immunized with the high dose mRNA/LNP vaccine. **(C)** Comparison of Gag Ab titers from the high and low dose mRNA/LNP regimens at two weeks after the 2^nd^ vaccination. **(D, E)** The antigen-specific cellular analysis was performed by flow cytometry at 2 weeks after the 2^nd^ vaccination. **(D)** Plot showing the Gag-specific CD4^+^ and CD8^+^ memory (CD3^+^CD95^+^IFN-γ^+^) T cell responses measured in PBMC. **(E)** Plot comparing the Gag-specific T cell responses as % of total T cells in macaques immunized twice with low dose (25 μg; described in **Figure 1**) and high dose (100 μg) mRNA/LNP vaccines, respectively. Responses in macaques immunized twice with 1 mg *gag* DNA (grey symbols) are included. The DNA vaccine contained IL-12 DNA as vaccine adjuvant and was administered by IM injection followed by electroporation. The p value is from t test (Mann-Whitney). **(F-G)** Analysis of gag mRNA/LNP vaccine induced memory CD4 immune responses. **(F)** Gating strategy for unstimulated and Gag peptide stimulated memory T cells producing IFN-γ and TNFα. **(G)** Pie charts showing responses of the 4 animals in the high dose vaccine group.

Macaque vaccine studies were conducted in compliance with all the state and federal regulations and were approved by BIOQUAL’s Institutional Animal Care and Use Committee (IACUC). The LNP-formulated RNActive vaccines encoding for HIV CE (p24CE) (16, 29) or *gag* (p55^gag^) (16, 29) are produced by CureVac AG, Tübingen, Germany, as detailed in (69). Lipid nanoparticle (LNP)-encapsulation of mRNA was performed by Acuitas Therapeutics (Vancouver, Canada). The macaque studies are detailed in **Table S1**. Fifteen naïve Indian rhesus macaques were enrolled in mRNA/LNP vaccination studies using 25 μg of mRNA in each vaccination. The animals were divided in three vaccine groups (n=5) with equal distribution of age, weight, and gender. All animals received 4 mRNA/LNP vaccinations by intramuscular injection onto the inner thigh at 0, 4, 12 and 24 weeks. Animals in group 1 were immunized with the CE mRNA/LNP vaccine only. Animals in group 2 received the *gag* mRNA/LNP only. Animals in group 3 were immunized twice with the CE mRNA/LNP vaccine followed by two vaccinations using a combination of the CE and *gag* mRNA/LNP vaccine. In a second study, five naïve animals were immunized twice (four weeks apart) with a 100 μg/dose of *gag* mRNA/LNP. Booster vaccine studies of animals previously immunized with plasmid DNA were performed with 25 μg dose of the *gag* mRNA/LNP vaccine. Priming or booster vaccinations with *gag* DNA were performed by intramuscular injection onto the inner thighs followed by *in vivo* electroporation using the Cellectra 5P device (Inovio Pharmaceuticals, Inc) as described (31, 70). The DNA p55^g^ *^ag^* (plasmid 114H) and p24CE (plasmid 306H) expressed Gag and CE, respectively, from codon-optimized sequences inserted between the human cytomegalovirus (CMV) promoter and the bovine growth hormone (BGH) polyadenylation signal (29, 30). The DNA dose was: 4 mg (**Figures 1F, G**), reported in (30); 1 mg (**Figure 2**), reported in (29); and 2 mg (**Figures 5**, **6**), detailed in **Table S1**. No significant differences between the 1 and 2 mg DNA dose using electroporation were found in our previous studies. The vaccines also contained 0.2 mg IL-12 DNA, except for the 5 DNA primed macaques shown in **Figures 5** (prime) and 6 (mRNA/LNP boost), detailed in **Table S1**.

### Cellular immune responses

PBMC were isolated from EDTA-blood by Ficoll-Paque™Plus (GE Health #17-1440-03) gradient centrifugation. Live-frozen cells were thawed, DNaseI-treated and counted upon Acridine Orange/Ethidium Bromide dye staining, detailed in (71). The viability typically was >90%. Bronchioalveolar lavage (BAL) was collected in by perfusion with PBS, and cells were collected after centrifugation and washing, and the cells were directly assayed. In both, PBMC and BAL, antigen-specific T cell responses were measured in peptide-stimulated cells, in the presence of the protein secretion inhibitor Monensin (GolgiStop), as previously described (30, 71). Briefly, 10^6^ cells were seeded in 96-well plates and stimulated with different peptide pools at a final concentration of 1ug/ml for each individual peptide. For negative and positive controls, cells were cultured in medium without peptides or stimulated with a PMA-cell stimulation cocktail (eBioscience, Affymetrix Inc. San Diego, CA, USA). PMA (Phorbol 12-Myristate 13-Acetate) stimulation was performed as described (71), and the median value obtained was 36% (range 22-46) of IFN-γ^+^ T cells. After 12 hours incubation, the cells were washed and stained with antibody cocktails targeting surface proteins. After 20 minutes of incubation, the cells were washed and fixed/permeabilized at 4°C in the dark using the FoxP3 fixation/permeabilization buffer (eBioscience, Affymetrix Inc. San Diego, CA). After washing the cells with permeabilization buffer (eBioscience by Affymetrix Inc.), the cells were stained with an antibody mix targeting cytokines and intracellular proteins. After 30 minutes of incubation at room temperature, the samples were washed, resuspended in PBS and acquired on a Fortessa or BDSymphony flow cytometer (BD Biosciences, San Jose, CA). The flow data were analyzed using FlowJo software (BD Biosciences, San Jose, CA). The following antibodies (clones in parenthesis) were used in these studies: CD3 (SP34-2), CD4 (L200), CD95 (DX2), CD69 (FN50), IFNγ (B27), TNFα (Mab11) (BD Biosciences); CD8 (RPA-T8), CD28 (CD28-2), CD137 (4B4-1) (Biolegend); CD107a (eBioH4A3), T-bet (4B10) (ThermoFisher/eBioscience). Gating strategies for PBMC and BAL lymphocytes are outlined in **Figures S1, S2A**, respectively. All flow data are shown after subtraction of the respective background values obtained after incubation of cells with medium only. The median values of the medium-only, unstimulated PBMC were 0.03 (IQR 0.07) for total CD3^+^, 0.019 (IQR 0.03) for CD4^+^ memory and 0.007 (IQR 0.02) for CD8^+^ memory T cell subsets. The median values of the medium-only, unstimulated BAL lymphocytes were 0.53 (IQR 0.53) for CD4^+^ memory and 1.01 (IQR 1.22) for CD8^+^ memory T cell subsets. To be considered positive, a BAL sample had to be at least 2-fold higher than the medium control and score above 0.05%.

### Humoral immune responses

Anti-p24^Gag^ antibodies were measured by ELISA using eight 4-fold serial dilutions of plasma samples, starting at 1:50 dilution. The OD450 measurements of the diluted samples were plotted, and GraphPad Prism area-under-the-curve was used to determine the endpoint titers above the baseline using the last X feature. Linear endpoint titers were used for comparative analysis.

### Cytokine measurements

Plasma samples, collected at the day of vaccination (day 1), and at day 2, 4, and 8 after each mRNA/LNP vaccination, were monitored using a U-PLEX Non-Human Primate Biomarker Assay (Meso Scale Diagnostics, MD, USA) for changes in the concentration of 61 cytokines/chemokines according to the manufacturer’s instructions (**Table S2**). Similar measurements were performed after a DNA-only vaccination.

### Bioinformatics and statistical analysis

The biomarker analysis was performed with a workflow written in R and through a user interface developed on the Foundry Platform (Palantir Technologies). Briefly, biomarkers falling below the detection limit/standard range were removed if absent in more than 50% of the samples or adjusted to 0.5 detection limit/standard point. The limma R package (v3.38.3) was used to compare biomarker changes between timepoints and R (v3.5.1) as implemented on the NIH Integrated Data Analysis Platform. (Link: https://github.com/NCI-VB/felber_curevac). Comparative analysis of immune responses was performed by paired or unpaired non-parametric t test using GraphPad Prism Version 9.2 for MacOS X (GraphPad Software, Inc, La Jolla, CA). p values are given only if significant statistical differences were found.

## Results

### HIV CE and Gag mRNA/LNP vaccination in macaques

A cohort of 15 naïve rhesus macaques (5 per group) was vaccinated with 25 µg doses of HIV mRNA/lipid nanoparticle (LNP). Group 1 was vaccinated with HIV mRNAs expressing conserved elements in p24^Gag^ (CE), a bivalent immunogen comprising of CE1 and CE2 differing by 7 amino acids to cover >98% of group M Gag, spanning 25% of Gag; group 2 was vaccinated with mRNA expressing the complete p55^Gag^ (gag), while group 3 was vaccinated with mRNA expressing a combination of CE prime followed by CE+Gag (12.5 µg each) boost (CE+gag) (**Figure 1A**). The same protocol was previously tested in macaques using the DNA platform (30) and is currently being tested in clinical trials (NCT03181789; NCT03560258; NCT04357821). As comparison, we tested each vaccine component (CE; gag) by itself.

Vaccinations with the mRNA/LNP formulations were safe in rhesus macaques. Some animals had mildly elevated body temperature (>1°F) 24 hours after vaccine delivery (**Figure S3**). This effect was transient, and the body temperature returned to normal levels within 3 days. No other significant side effects were observed either systemically or at the injection site (intramuscular delivery in the quadriceps).

Anti-Gag antibodies (Ab) were detected in all the animals after the 2^nd^ vaccination (**Figure 1B**), reaching peak responses after the 3^rd^ vaccination in all groups, irrespective of the immunogen used. Responses to the vaccinations were rapid and reached maximal levels one to two weeks after each vaccination. Ab levels showed similar peak responses for the CE (group 1) and gag group (group 2), in agreement with our previous observations with DNA vaccinations (15). There was no difference among the groups up to 8 weeks post vaccination 4 (week 32). The Ab responses were further monitored over time in a subset of 8 macaques (groups 1 and 3). We found sustained Gag Ab responses (**Figure 1C**) with a bi-phasic decline with an initial median 2.4 log decline to week 40 which then plateaued up to week 62 of the follow-up. Together, the data illustrate the induction of robust and durable Ab responses by the CE and CE/CE+Gag mRNA/LNP vaccinations.

Vaccine-induced antigen-specific T cell responses were analyzed in PBMC by flow cytometry upon stimulation with p55^Gag^ and CE peptide pools using the gating strategy outline in **Figures S1** at 2 weeks after the 4^th^ vaccination. Threshold levels of responses were found after 2 vaccinations, while T cell memory (CD28^+^CD95^+^ and CD95^+^CD28^-^) responses to Gag (**Figure 1D**) and CE (**Figure 1E**) were detected in the majority of the animals at 2 weeks after the 4^th^ vaccination. The response rate for the vaccine-induced T cell immunity was less consistent among animals in the different groups, than the strong humoral responses elicited by the vaccines in all macaques (**Figure 1B**). Gag- and CE-specific T cell responses were mediated by both CD4^+^ and CD8^+^ memory T cells (**Figures 1D, E**, upper and lower panels). The antigen-specific CD4^+^ T cell responses were compatible with Th1 phenotype (IFN-γ and TNF-α secretion) (see also **Figure 2G**). The animal-to-animal difference in ability to mount distinct (CD4 vs CD8) T cell responses was as expected from outbred macaques. Importantly, despite the overall low level of antigen-specific IFN-γ^+^ CD4^+^ T cell responses in blood, we found remarkable durability of humoral responses (**Figure 1C**), supporting the presence of efficient CD4^+^ T helper responses. As we reported previously (70, 72–75), there was a greater animal-to-animal variation in cellular responses compared to humoral responses, found also in these outbred macaques

Studies from us and others have shown that DNA vaccination, using an optimized formula including IL-12 DNA as vaccine enhancer, administered by IM injection followed by electroporation induced robust [~4x higher (76)] antigen-specific T cells [reviewed in (33, 38)]. Therefore, the T cell responses elicited by the mRNA/LNP vaccine regimen in group 3 were compared to responses obtained from macaques vaccinated with DNA (4 mg dose) expressing the same immunogens [CE prime-CE+gag boost (30)]. This comparison showed lower T cell responses in the mRNA/LNP group targeting Gag (~40-fold, **Figure 1F**, left panel) and CE (~80-fold, **Figure 1F**, right panel) epitopes. It is possible that differences in the dose between the mRNA vs DNA vaccines (20-times more molecules of DNA than mRNA, although the respective efficiency of *in vivo* transduction and expression of DNA versus mRNA is unknown) or the vaccine composition contributed to this.

Comparison of vaccine-induced Gag Ab levels in the matching DNA group showed similar kinetics over time (**Figure S4**) as the mRNA/LNP group with similar durability (**Figure 1G**). The *gag* mRNA/LNP group could not be further assessed because the animals were reassigned to the mRNA/LNP booster study (see **Table S1**, **Figure 5**). Like the mRNA vaccine, the DNA vaccine induced Gag Ab that showed a biphasic decline with an initial median 1.8 log decline over ~25 weeks, which then plateaued up to week 73 of the follow-up. Long-term durability showed similar sustained Ab levels upon the 4^th^ vaccination in the mRNA (week 62) and DNA (week 73) groups (**Figure 1H**).

In conclusion, the HIV mRNA/LNP vaccines induced high durable humoral but low cellular responses, even after 4 vaccinations, in naïve vaccinated macaques. The analogous DNA vaccine induced similar levels of humoral responses but significantly higher cellular responses.

### High dose *gag* mRNA/LNP vaccine in naïve macaques

We tested whether increasing the mRNA/LNP dose from 25 to 100 μg could improve the induction of antigen-specific immune responses (**Figure 2**). Macaques (n=5) were immunized twice with a 100 μg dose of the *gag* mRNA/LNP vaccine (**Figure 2A**). After the 2^nd^ vaccination, the immune responses were compared to data from animals in group 2 (see **Figure 1**), vaccinated twice with the 25 μg dose.

Anti-Gag Ab were detected in all five animals (**Figure 2B**) after the 2^nd^ vaccination. Comparison to the 25 μg dose (**Figure 2C**) showed only a slightly higher level (median 2.7 fold) which did not reach significance. These data indicated that increasing the vaccine dose did not provide an additional advantage for the development of humoral responses.

Analysis of the Gag-specific T cells revealed induction of both CD4^+^ and CD8^+^ Gag-specific memory T cell responses (**Figure 2D**). A higher response rate (4 of 5 macaques) was found compared to the low-dose group after the 2^nd^ vaccination (**Figure 2E**). Total Gag-specific T cell responses were ~6-fold higher in the 100 μg dose group, reaching up to 0.12% of Gag-specific IFN-γ^+^ T cells. These levels were still significantly lower (median 10-fold; p=0.008, Mann-Whitney) than those obtained upon two *gag* DNA vaccinations (1 mg dose with IL-12 DNA) administered by IM injection followed by electroporation (**Figure 2E**). In addition to IFN-γ production, we further evaluated the Th1 phenotype of the antigen-specific CD4^+^ memory T cells including TNFα secretion (**Figure 2F**). The pie charts show the induction of IFN-γ^+^, TNFα^+^ and IFN-γ^+^ TNFα^+^ Gag-specific T cell responses (**Figure 2G**).

Together, vaccination with high dose *gag* mRNA/LNP vaccines induced similar levels of humoral immune responses but resulted in increased magnitude of cellular immune response in comparison to the low-dose vaccination.

To address sequestering of the mRNA/LNP induced T cell responses, we performed analysis of lymphocytes collected from bronchioalveolar lavage (BAL), serving as surrogate for T cell responses found at mucosal sites. The analysis was performed at 2 weeks after the 3^rd^ vaccination of mRNA/LNP vaccinated macaques shown in **Figure 1** and at 2 weeks after the 2^nd^ vaccination of macaques shown in **Figure 2**. The gating strategy (**Supplementary Figure 2A**) and the presence of antigen-specific IFN-γ^+^ and TNFα^+^ effector (CD28^-^, CD95^+^) and transitory (CD28^+^, CD95^+^) memory T cells (**Figure S2B**) are shown. These data demonstrate that IM delivery of mRNA/LNP vaccine is able to induce antigen-specific T cell responses that disseminate to mucosal tissue, i.e. lung, as we have previously shown for the DNA vaccine induced immune responses (73, 77).

### Changes in plasma cytokine levels after mRNA/LNP vaccination

**Figure 3:**
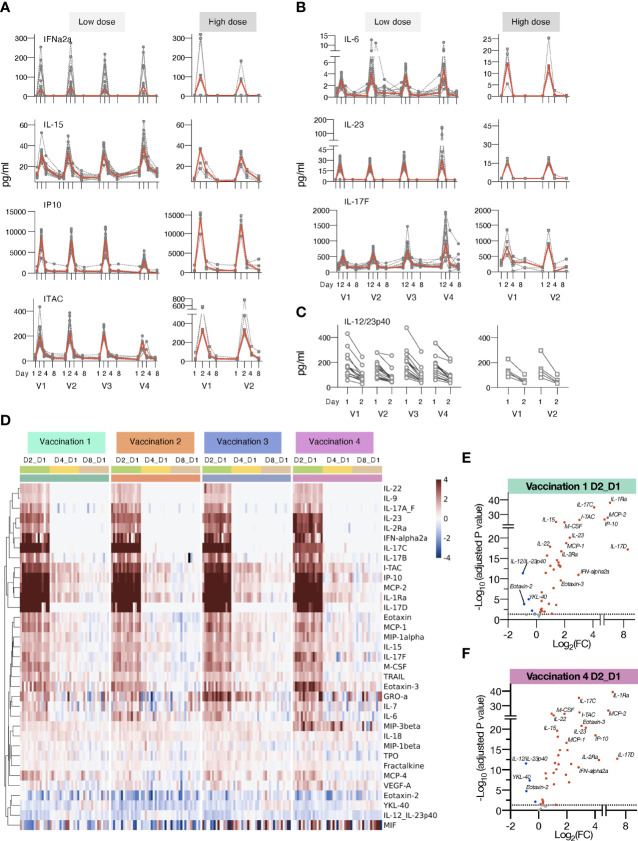
Changes in plasma cytokines after vaccination with mRNA/LNPs. Plasma cytokine and chemokine levels were measured using the MSD assay on the day of (D1) and days 2, 4 and 8 (D2, D4, and D8) after each vaccination in macaques receiving mRNA/LNPs vaccine. **(A, B)** Circulating plasma levels of selected analytes for individual animals (grey lines) and median (red lines) are shown upon the mRNA/LNP vaccinations, administered with low (25 μg, left panels) or high (100 μg, right panels) dose. **(A)** Molecules involved in IFN pathway, IFNα-2a, IL-15, IP-10/CXCL10, and ITAC/CXCL11. **(B)** Molecules involved in the IL-17 pathway, IL-23, IL-6, and IL-17F. **(C)** Decay in the circulating plasma levels of IL-12/23p40 between D1 and D2 for the individual animals upon each mRNA/LNP vaccination after receiving low dose (left panel) or high dose (right panel) mRNA/LNP vaccine. **(D)** Heatmap depicts log2 fold changes (log2 FC) in 35 analytes overtime upon each vaccination (light green: D2_D1; yellow: D4_D1; tan: D8_D1). Cytokine levels at D1 before each vaccination are used as baseline. Comparisons were performed between day1 and day 2 (D2), day 4 (D4) and day 8 (D8), respectively, with data for each animal shown under colored with vaccination 1 to 4 are indicated by the green, orange, blue, and purple bars, respectively. **(E, F)** Volcano plots of data shown in panel D depict differentially expressed analytes upon the vaccination 1 **(E)** and vaccination 4 **(F)** at day 2 versus day 1. Red dots indicate significant upregulation; blue dots indicate significant downregulation (adjusted p value<0.05 represented by the broken horizontal line).

**Figure 4:**
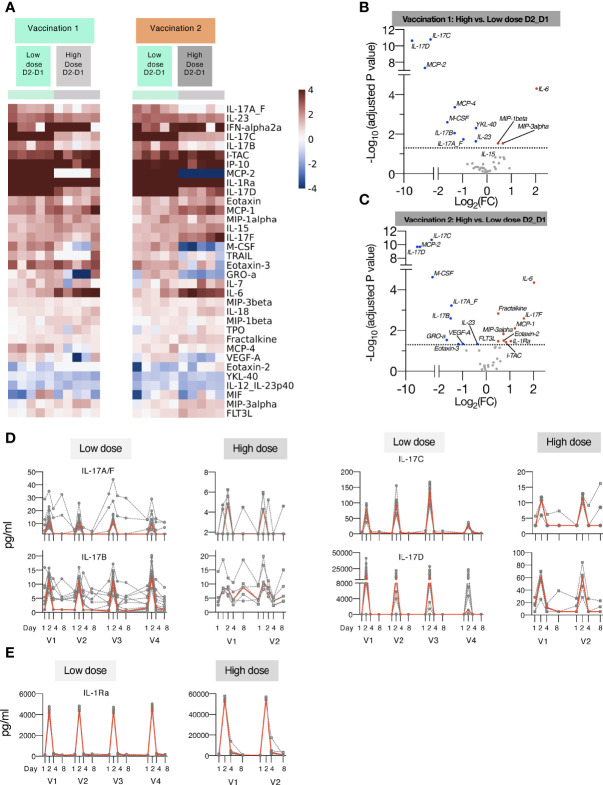
Comparison of cytokine and chemokine levels measured in macaques upon low and high dose mRNA/LNP vaccinations. Plasma cytokine and chemokine levels were measured using the MSD assay in macaques after the 1^st^ and 2^nd^ mRNA vaccine doses, administered at low (green) or high (grey) mRNA/LNP doses. **(A)** Heatmap depicts log2 fold changes in 34 analytes detected at 24 hours (D2_D1) after Vaccination 1 (green) and Vaccination 2 (orange). Cytokine levels at D1 before each vaccination are used as baseline. **(B, C)** Volcano plots of data shown in panel A depict differentially induced changes upon Vaccination 1 **(B)** and Vaccination 2 **(C)** between low and high mRNA vaccine doses. Red dots indicate analytes significantly more upregulated in animals receiving the high dose vaccine; blue dots indicate analytes significantly more upregulated in animals receiving the low dose vaccine (adjusted p value<0.05 represented by the broken horizontal line). **(D, E)** Overtime changes in inflammatory modulators upon mRNA/LNPs vaccination. Circulating plasma levels of **(D)** IL-17 family of cytokines (IL-17A/F, IL-17B, IL-17C, IL-17D) and **(E)** IL-1Ra for individual animals (grey lines) and median (red lines) are shown upon mRNA vaccination, administered at low (left panels) and high (right panels) mRNA/LNP doses.

We investigated the cytokine signature induced by the mRNA/LNPs vaccination in the macaques shown in **Figures 1**, **2** (25 and 100 µg/dose). Plasma was collected at the day of vaccination (Day 1) and over time (Days 2, 4 and 8) after each vaccination, and cytokine analysis was performed using the MSD (Meso Scale Discovery) platform. We evaluated the plasma levels of the 61 analytes listed in **Table S2**. The cytokine and chemokine profiles measured overtime after each vaccination were represented in heatmaps, volcano plots and plots of selected analytes (**Figures 3**, **4**, **S5**). No difference was found among the three low-dose vaccine groups (described in **Figure 1**), therefore individual measurements were combined for the subsequent analysis of the 15 animals and were also compared to the 5 animals (described in **Figure 2**) that received the high-dose mRNA/LNP vaccine.

The low-dose mRNA/LNP vaccinations were associated with a rapid up-regulation (24 hrs post vaccine administration, D2) of type I IFN (IFN-α2a), IL-15, a cytokine involved in the expansion/survival of cytotoxic memory lymphocytes and NK cells (reviewed in [78)], and IFN-responsive chemokines, such as IP-10/CXCL10 and ITAC/CXCL11 (**Figures 3A, D**). A rapid induction of the pro-inflammatory cytokines IL-6 and IL-23 was also observed after each vaccination. This systemic response resulted in the release of different members of the IL-17 family of cytokines (**Figures 3B, D**, **4D**), as previously reported (79–81). Down-stream of the immunological IL-23/IL-17 axis are other pro-inflammatory cytokines like IL-22 (82), MCP-1/CCL2 and GROa/CXCL1 (83) which were also increased after each vaccination (**Figure 3D**). All mRNA/LNP vaccinations resulted in decreased plasma levels of IL-12/23p40 (**Figures 3C, D**), the common chain for the heterodimeric IL-12 and IL-23. This decrease was observed within 24 hours of vaccination irrespective of the mRNA/LNP dose (**Figure 3C**). IL-12p70 was also monitored as part of the MSD assay, but its plasma concentration was below the limit of detection.

IL-1Ra, a cytokine with an anti-inflammatory role, was also increased (**Figures 3D**, **4E**), as were several other chemokines, such as MIP-3β/CCL19, Eotaxin/CCL11, Eotaxin-3/CCL26, MCP-1/CCL2) and MIP-1α/CCL3, responsible for the recruitment of lymphoid and myeloid cells (**Figure 3D**). Cytokine levels peaked on the days after vaccinations and some of the effects induced by vaccination were still detectable at day 4, with persistent elevated levels of chemokines including IP-10/CXCL10, ITAC/CXCL11, MCP-2, MIP-3β/CCL19, and inflammatory modulators IL-18 and IL-1Ra, which declined to baseline by day 8. The circulating levels of all the affected cytokines returned to baseline by day 8 post vaccination (**Figure 3D**).

Subsequent vaccinations resulted in an overall similar cytokine profile, suggesting that the innate responses to the mRNA/LNP vaccine mainly affected the observed signature. Notably, the response magnitude for some analytes (e.g. IP-10/CXCL10, ITAC/CXCL11, IL-17C, IL-17D) was reduced after the 4^th^ vaccination (**Figures 3**, **4D**).

We also performed differential expression analysis comparing mean log2 fold change (Log2FC) of cytokine levels at day 2 to day 1 for all the 15 animals (shown in **Figure 1**) receiving the low dose mRNA/LNP vaccine (**Figures 3E, F**, **S5**). The cut-off was set at p<0.05 and was adjusted for multiple comparisons. All vaccinations resulted in a cytokine profile featuring several inflammatory modulators, such as cytokines and chemokines related to the IFN and the IL-17 pathways. The mRNA/LNPs vaccines also negatively impacted the levels of IL-12/23p40, Eotaxin-2 and YKL-40.

Additionally, increasing the vaccine dose showed overall a similar pattern of cytokine/chemokine induction a depicted in heatmaps after the 1^st^ and 2^nd^ vaccination (**Figure 4A**) and in volcano plots (**Figures 4B, C**). We noted distinct changes in the response magnitude with analytes being lower (e.g., some members of IL-17 family, IL-23) or higher (e.g., IL-6, IL-1Ra, ITAC/CXCL11). The high dose vaccination was associated with significant higher plasma levels of IL-6 and the chemokines MIP-1β, MIP-3α and ITAC/CXCL11, indicative of the induction of a stronger inflammatory response. Concomitantly, circulating levels of IL-1Ra were ~10-fold higher in macaques receiving the high dose vaccine in comparison to low dose (**Figure 4D**). On contrary, the high vaccine dose was associated with reduced serum levels of IL-23, IL-17A_F, IL-17B, IL-17C, IL-17D (**Figures 3B**, **4D**), and monocyte/macrophage chemoattractant M-CSF, MCP-2, MCP-4 (**Figures 4A-C**). Both the high and low dose mRNA/LNP vaccines negatively impacted the levels of IL-12/23p40, YKL-40 and MIF (**Figures 3**, **4**). The changes in cytokine/chemokine levels did not correlate with changes in immune responses but rather reflected innate activation triggered by the chemical composition of the mRNA/LNP. We did not test the effect of LNP only.

**Figure 5:**
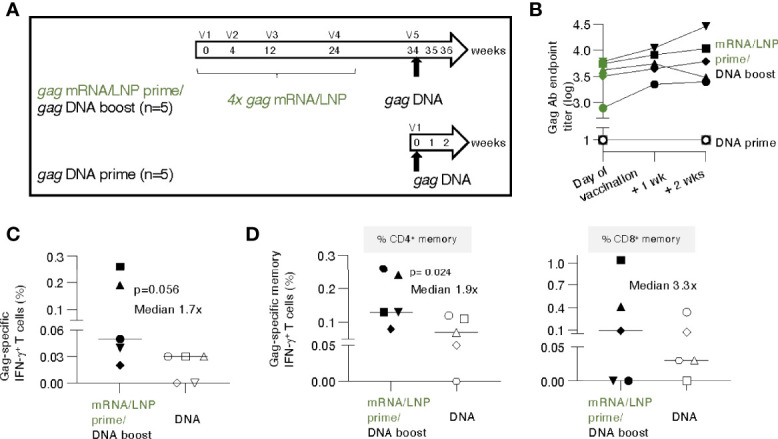
gag DNA booster vaccination of macaques primed with mRNA/LNP vaccinations increased T cell responses. **(A)** Schematic representation of the mRNA/LNP prime - DNA boost vaccination regimen. Five animals previously immunized four times (V1-V4) with *gag* mRNA/LNP (group 2, 25 μg dose, described in **Figure 1**) received a single *gag* DNA vaccination (V5; 2 mg dose) at 10 weeks after the last mRNA/LNP vaccination (V4). A group of naïve macaques (n=5) received a single *gag* DNA vaccination and was added for comparison. The *gag* DNA vaccine was administered by IM injection followed by electroporation. **(B)** Gag Ab titers after the single DNA vaccination were plotted over time. **(C, D)** Gag-specific cellular analysis was performed by flow cytometry two weeks after the DNA vaccination. **(C)** Total Gag-specific (CD3^+^IFN-γ^+^) T cell responses as % of total T cells and **(D)** Gag-specific memory (CD3^+^CD95^+^IFN-γ^+^) T cell responses are shown. The percent of Gag-specific IFN-γ^+^ CD4^+^ (left panel) and CD8^+^ (right panel) memory T cells in blood were plotted. The p values are from t test (Mann-Whitney).

In contrast to the responses found upon mRNA/LNP vaccination, the responses upon a single *gag* DNA only vaccination administered by electroporation (5 animals shown in **Figure 5**), showed delayed chemokine/cytokine responses, typically reaching peak by days 4-8 post vaccination and lower peak values compared to the mRNA/LNP vaccine. Increase was detected only for a subset of analytes including IL-1Ra (~50x lower than mRNA/LNP), CXCL10/IP10 (~15x lower), IL-15 (5x lower), CXCL9/ITAC (~6x lower). These data indicate profound differences in the innate immune responses between the IM delivered mRNA/LNP and DNA vaccines.

Overall, these data identified a cytokine signature induced by the mRNA/LNP vaccine characterized by the induction of inflammation and recruitment of immune cells (both lymphoid and myeloid cells).

### DNA booster vaccination of the T cell responses primed by *gag* mRNA/LNP vaccination

Next, we evaluated whether the immune responses elicited by the *gag* mRNA/LNP vaccination could be boosted by a subsequent single *gag* DNA vaccination. The study was designed to evaluate the initial response to a heterologous booster vaccination, i.e., using a single *gag* DNA immunization. The concept of a DNA booster for immune responses induced by mRNA/LNP vaccination was used in lieu of HIV infection-induced responses that cannot be tested in macaques.

Animals from group 2 (see **Figure 1**), which had received prior 4 *gag* mRNA/LNP vaccinations, were subjected to a single *gag* DNA booster vaccination (2 mg dose without IL-12 DNA to parallel mRNA/LNP vaccine which did not contain IL-12), administered by electroporation, after a 10-week rest (**Figure 5A**). To distinguish recall versus *de novo* responses, five gag-naïve macaques receiving a single DNA vaccination, were included as controls.

The *gag* mRNA/LNP vaccinated animals showed high levels of Gag antibodies (median 3.6 log, range 2.9-3.8) on the day of vaccination and elicited rapid, anamnestic responses upon a single *gag* DNA administration with a modest median increase (0.3 log, range 0.1-0.7) (**Figure 5B**) over the relatively high pre-existing levels. In contrast, a single *gag* DNA vaccination of naive macaques did not induce detectable humoral responses within the 2 weeks of follow-up (**Figure 5B**). These data showed that mRNA/LNP primed humoral responses could be boosted by a DNA vaccination.

T cell responses were analyzed at 2 weeks post DNA vaccination. Comparison of Gag-specific T cell responses showed a higher response rate and a trend of higher magnitude in the group with pre-existing immunity (**Figure 5C**). The Gag-specific responses were significantly higher among the CD4^+^ memory subset (**Figure 5D**, left panel; median 0.13% versus 0.07%), likely reflecting their priming with the prior mRNA/LNP vaccination. The difference in CD8^+^ memory responses (median 0.1% versus 0.03%) did not reach significance (**Figure 5D**, right panel). Comparison to the magnitude reached at peak upon the 4^th^ mRNA/LNP vaccination only (see **Figure 1D**) showed a further increase of T cell memory responses (CD4^+^ increase: median 0.08% to 0.13%; CD8^+^ increase: 0.06% to 0.1%) after the *gag* DNA boost.

These data show that the low pre-existing T cell responses induced by the mRNA/LNP vaccine were modestly boosted after a single DNA vaccination. The magnitude of antigen-specific memory CD4^+^ T cells upon one single DNA vaccination was significantly higher in macaques previously immunized with mRNA/LNP compared to naïve animals, which supports the concept of an anamnestic response due to priming by the *gag* mRNA/LNP vaccination.

### *Gag* mRNA/LNP vaccine boosts pre-existing humoral and cellular immunity induced by *gag* DNA vaccination

**Figure 6:**
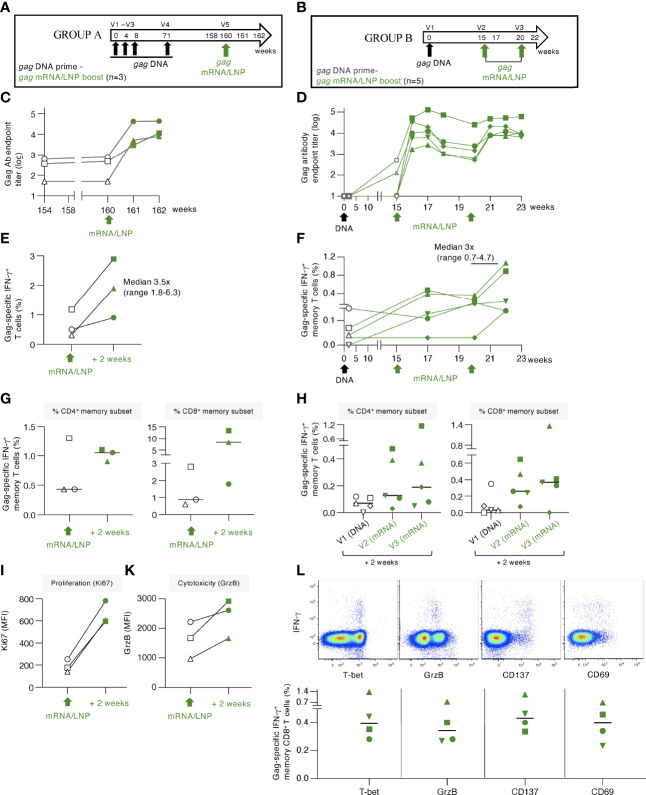
gag mRNA/LNP booster vaccination of macaques with pre-existing Gag T cell immunity increased T cell responses. **(A, B)** Schematic representations of the DNA prime - mRNA/LNP booster vaccination regimens. **(A)** The macaques (n=3) in group A previously received 4 HIV *gag* DNA vaccinations (week 0, 4, 8 and 71). After a rest period of 89 weeks, they received a single *gag* mRNA/LNP (25 μg) booster vaccination (V5). **(B)** Animals in group B received a single *gag* DNA prime (V1; 2 mg dose), followed 15 weeks later by two *gag* mRNA/LNP booster vaccinations (V2, V3; 25 μg dose) spaced 5 weeks apart. **(C, D)** Gag-specific Ab endpoint titers (log) were measured by ELISA during the course of the studies. **(C)** Gag Ab were measured starting 6 weeks before study start (week 154), at the day of vaccination (week 160), and 2 and 4 weeks upon the mRNA/LNP boost. **(D)** Gag Ab responses were measured after the *gag* DNA vaccination, at the start and post the mRNA/LNP vaccinations. **(E, F)** Gag-specific T cell responses measured by flow cytometry at the indicated timepoints for **(E)** group A and **(F)** group **(B)** Grey symbols denote responses after the DNA vaccination, green symbols denote responses after mRNA/LNP vaccination. **(G, H)** Gag-specific responses in total (CD3^+^IFN-γ^+^) and memory (CD3^+^CD95^+^IFN-γ^+^) T cell subsets are shown. Changes in **(I)** proliferation, measured by Ki67 staining, and **(K)** cytotoxicity, measured by granzyme B content, are shown for animals from group A. **(L**) Dot plots (upper panels) from a representative animal (LI19) from group B showing T-bet, granzyme B content and expression of the co-stimulatory immune checkpoint molecule CD137 and the CD69 activation marker among the Gag-specific IFN-γ^+^ memory CD8^+^ T cells after the last vaccination. The graph (lower panel) shows the peak responses after the last vaccination with data from 4 of 5 animals with positive Gag-specific memory (CD8^+^CD95^+^IFN-γ^+^) T cell responses.

We next examined the impact of a *gag* mRNA/LNP booster (**Figure 6**) for animals with different levels (high, group A (**Figure 6A**) or low, group B (**Figure 6B**) of pre-existing immunity. Since macaques cannot be infected by HIV, we selected DNA vaccinated macaques to serve as model for pre-existing immunity. Animals in group A had previously received four HIV *gag* DNA vaccinations over a period of 3 years (**Figure 6A**, n=3), and after a rest of 89 weeks, had a median anti-Gag antibody titer of 2.6 log (range 2.2-2.9 log) at week 158, prior to the mRNA vaccination (**Figure 6C**). Inclusion of IL-12 DNA adjuvant in the priming vaccination ~1.5 years prior predicted not thought to have any effect on the mRNA/LNP boosting in these animals. Animals in group B had received a single HIV *gag* DNA vaccination (**Figure 6B**, n=5) and, after a 15-week rest, their Gag Ab levels were low with only two animals showing titers (2 and 2.7 log, respectively) above the threshold of the assay (**Figure 6D**) (see also **Table S1**).

Administration of a single *gag* mRNA/LNP vaccination in group A resulted in a sharp increase (median of 1.7 log) of Gag Ab titers (**Figure 6C**). Similarly, a single *gag* mRNA/LNP booster vaccination in group B resulted in rapid anamnestic humoral response, reaching up to 5 log of anti-Gag Ab titer (**Figure 6D**). The antibody response in group B showed a slight contraction 3-5 weeks later and was efficiently boosted again by a 2^nd^ mRNA/LNP vaccination, reaching similar peak Ab levels. Together, the data shown in **Figures 6C, D**, demonstrated that a single *gag* mRNA/LNP vaccination was able to induce robust anamnestic humoral responses independent of the magnitude of pre-existing immunity.

Gag-specific T cell responses induced in these two groups of animals were analyzed in PBMC (**Figures 6E, F**, respectively). In group A, the priming DNA vaccinations induced Gag-specific T cells that were still detectable 89 weeks after the last vaccination (range 0.3-1.2% of T cells). A single mRNA/LNP vaccination efficiently boosted these responses (2- to 6-fold) in all 3 animals reaching up to 3% of circulating T cells (**Figure 6E**). Analysis of the pre-existing memory responses showed ranges of 0.4-1.3% CD4^+^ and 0.6-2.8% CD8^+^ memory T cells (**Figure 6G**). Two animals showed increases of Gag-specific CD4^+^ and CD8^+^ T cells and one animal showed increase only in CD8^+^ T cells. The responses reached levels up to 1.1% CD4^+^ and 13.4% memory CD8^+^ T cells in blood. Characterization of the boosted Gag-specific T cells showed a phenotype of activated cytotoxic T lymphocytes (CTL) with increased proliferative capacity measured by Ki67 expression (**Figure 6I**) and increased granzyme B (GrzB) content (**Figure 6K**).

Administration of *gag* mRNA/LNP booster vaccination in animals of group B (**Figure 6B**) was also successful in stimulating low pre-existing T cell responses (**Figure 6F**). Gag-specific T cell responses increased in all five macaques, with three animals showing responses after the 1^st^ vaccination, and all five animals showing increase after the 2^nd^ mRNA/LNP booster vaccination. The boosted responses were mediated by both CD4^+^ and CD8^+^ Gag-specific T cells, with a dominant CD8 response (**Figure 6H**). The antigen-specific IFN-γ^+^ CD8^+^ T cell responses in both groups were characterized by the expression of T-bet and GrzB, reminiscent of a cytotoxic memory phenotype, and the activation markers CD137 and CD69 (**Figure 6L**).

Importantly, the *gag* mRNA/LNP vaccine was more powerful as booster for recall (administered a single time) of cellular immune responses (**Figure 6**) than for inducing *de novo* T cell responses (administered 4 times) (**Figures 1**, **2**). Therefore, the very effective boosting of pre-existing T cell immunity by the HIV *gag* mRNA/LNP could have general application of this vaccine platform as part of prime-boost regimen. Thus, a heterologous prime/boost regimen aiming to elicit balanced humoral and cellular immunity might be achieved by DNA (or i.e., infection-induced) prime-mRNA boost vaccination.

## Discussion

In this study, we show that HIV-1 *gag* mRNA/LNP vaccine regimens induced high antibody responses reaching maximal levels after the 3^rd^ vaccination but were less efficient in the induction of primary T cell responses in naïve rhesus macaques. This dichotomy has already been noticed with other mRNA-based vaccines in certain studies reporting low antigen-specific T cell responses in blood of macaques and humans (35, 55, 57, 59, 60, 63, 64, 84). Although induction of adaptive T cell responses by our CE/gag mRNA/LNP vaccine was low in naïve macaques in comparison to a DNA vaccine regimen, we found persistence and similar magnitude of Gag antibody responses for >62 weeks after the 4^th^ vaccination. These data indicate that despite low levels of the antigen-specific IFN-γ^+^ CD4^+^ T cells in the blood, our mRNA/LNP vaccine induced efficient CD4^+^ T helper responses, enabling extended longevity of the humoral responses. It is possible that further modification of the mRNA/LNP vaccine components or inclusion of IL-12 as adjuvant could promote stronger T cell responses.

In contrast to *de novo* responses, the mRNA/LNP booster vaccination of animals with pre-existing Gag-specific T cells resulted in rapid recall (~3-fold increase) T cell responses. The induced T cells showed a Gag-specific cytotoxic effector phenotype characterized by high granzyme B content and T-bet expression, a transcriptional factor associated with Th1 response and cytotoxic CD8^+^ and NK cells (85). Our macaque studies showed rapid and high Ab responses upon a single *gag* mRNA/LNP booster vaccination, and these Ab responses were of higher magnitude than those elicited by a single low or high dose *gag* mRNA/LNP vaccination in naïve animals. By analogy, in humans, a single SARS-CoV-2 mRNA vaccination [BNT162b2 mRNA (41); CVnCoV (61)] also efficiently boosted antibodies in persons with pre-existing immunity, being more efficient than vaccination of COVID-19-naïve persons (41, 86). In addition, mRNA/LNP vaccination induced CD4^+^ T cell responses against SARS-CoV-2 more readily in convalescent patients (87). These data support the conclusion that heterologous vaccine regimens combining e.g., DNA with mRNA/LNPs could be a promising regimen to induce optimal, effective, and balanced humoral and cellular immunity. Specifically, the inclusion of mRNA-based immunogens could be useful in immune therapeutic regimens aiming to treat chronic HIV-1 infection or other pathological conditions to enhance pre-existing immunity.

Cytokines and chemokines are important drivers of inflammation and innate immunity and have a pivotal role in the development and maintenance of adaptive immunity in response to both infection and vaccination. The identification of a cytokine signature could be instrumental for vaccine optimization (88–91). Immune signatures have been reported in different vaccine studies in humans including Yellow fever, HIV-Ade5, HIV ALVAC, SARS-CoV-2 BNT162b2 mRNA (41, 92–95). To identify markers associated with vaccination with the *gag* mRNA/LNP, we studied cytokines and chemokines triggered by prime and boost vaccinations in macaques. We found that mRNA/LNP vaccinations triggered significant systemic transient (24 hrs) innate cytokine responses characterized by the release of type I IFN, IL-15 and interferon-related chemokines. We also observed a decrease in the plasma levels of IL-12/23p40 after each mRNA vaccination, but, in contrast, we found an increase in the IL-23 concentration, a cytokine that shares the p40 chain with IL-12. This increase, together with the increase in IL-6, resulted in repeated stimulation of several pro-inflammatory cytokines, especially those from the IL-17 family. The relationship between IL-23 and Th-17 cells is a well-known pro-inflammatory axis (79–81, 96) that is activated in several human diseases.

We had previously reported that SARS-CoV-2 BNT162b2 mRNA vaccine in human volunteers induced distinct early (24 hrs) transient cytokine responses featuring IL-15, IFN-γ and IP-10/CXCL10 that also included TNF-α and IL-6, upon booster vaccination (41). In addition, we had reported that the BNT162b2 mRNA vaccine-induced IFN-γ and IL-15 changes correlated with Spike-RBD antibody responses (41), associating these biomarkers with effective development of vaccine-induced humoral responses upon modified mRNA/LNP vaccination. In comparison to the human study, using a different mRNA vaccine platform in macaques, we also found significant increases of IL-15, IP-10/CXCL10 and IL-6, but the levels of critical components of the signature including IFN-γ and TNF-α were below the threshold of the assay in macaques. It is intriguing that two human vaccine studies with different platforms using BNT162b2 mRNA COVID-19 (41) and the non-replicating HIV-ALVAC vaccine [expressing HIV Gag, Pro, Env by a non-replicating avian vaccinia vector (canary pox virus) and alum-adjuvanted gp120 protein (94);] showed induction of cytokines IFN-γ, IL-15 and IP-10/CXCL10. Both IL-15 and IP-10/CXCL10 were also strongly induced upon *gag* mRNA/LNP vaccination in macaques. Both IFN-γ and IP-10/CXCL10 play a role in the IL-15 effects on the immune system (97–99) and a mechanism by which IL-15 indirectly acts on dendritic cells and macrophages/monocytes to induce the secretion of IP-10/CXCL10 *via* IFN-γ has been reported (100) [reviewed in (78)]. In contrast to the macaque study, the human study did not show detectable levels or changes for the IL-17 chemokine family and IL-23. The underlying reasons to explain such differences includes species (human, macaques); nature of mRNAs (modified versus non-chemically modified); immunogen (SARS-CoV-2 Spike versus HIV Gag based immunogen); and the statistical variation due to the small numbers of macaques enrolled (15 macaques versus 58 human volunteers). Thus, although the macaque study shared some of the chemokine/cytokine markers with the human study, it did not reveal a strong signature correlating to adaptive immune responses. In contrast to the human study with BNT162b2 mRNA which showed stronger innate responses upon the 2^nd^ vaccination (41), our macaque study showed comparable responses upon each vaccination, indicating key differences between the models.

In this report, we show that the *gag* mRNA/LNP vaccine induced high and durable antibody responses and low T cell responses in naïve macaques. In comparison, an antigen-matched DNA vaccine induced both strong antibody and T cell responses. Importantly, including a mRNA/LNP booster vaccination in DNA primed macaques augmented potent cytotoxic T cell responses, supporting the potency of the mRNA/LNP vaccine. Therefore, its application in a combination vaccine with other platforms including DNA or as a therapeutic vaccine to stimulate pre-existing immunity is promising.

## Data Availability Statement

The original contributions presented in the study are included in the article/**Supplementary Material**. Further inquiries can be directed to the corresponding author.

## Ethics Statement

The animal study was reviewed and approved by BIOQUAL’s Institutional Animal Care and Use Committee (IACUC).

## Author contributions

Conceptualization: SD, KC, LO, BP, EL, JM, AV, GP, BF. Data curation: AV, MR, CB, MAg, BF, GP. Formal analysis: AV, MR, CB, MAn, BF, GP. Investigation: AV, CB, MR, RB, MAg, JG, BF, GNP. Visualization: AV, MR, CB, MAn, BF. Writing – original draft: AV, CB, GP, BF. and Writing – review and editing: all coauthors. All authors contributed to the article and approved the submitted version.

## Funding

This work was supported with funds from the Intramural Research Program, National Institutes of Health, National Cancer Institute, Center for Cancer Research (GP, BF). This study received funding from National Institutes of Health award AI027757 (JM), and Inovio Pharmaceuticals Inc. under NCI CRADA#02289 (GP, BF). The funders were not involved in the study design, collection, analysis, interpretation of data, the writing of this article or the decision to submit it for publication. All authors declare no other competing interests.

## Acknowledgments

We thank D. Weiss, J. Treece, J. Misamore and staff (BIOQUAL, Inc.) for excellent support with the macaque studies; Y. Wang and J. Inglefield, Clinical Services Program, Frederick National Laboratory for Cancer Research for technical assistance; I. Kalisz (ABL, Inc.) for ELISA assays; X. Hu, Z. Lu, Y. Cai and V. Kulkarni for sharing data and members of the Felber and Pavlakis labs for discussions; T. Jones for editorial assistance. We thank Acuitas Therapeutics for LNP formulations of mRNA vaccines. All authors reviewed and edited the manuscript and gave final approval for the submitted version.

## Conflict of Interest

Authors JM, GP, BP and BF report issued patents of relevance to this work. Author MAn was employed by Leidos Biomedical Research, Inc. Authors JG, BP, LO, EL are employed by CureVac AG.

The remaining authors declare that the research was conducted in the absence of any commercial or financial relationships that could be construed as a potential conflict of interest.

## Publisher’s Note

All claims expressed in this article are solely those of the authors and do not necessarily represent those of their affiliated organizations, or those of the publisher, the editors and the reviewers. Any product that may be evaluated in this article, or claim that may be made by its manufacturer, is not guaranteed or endorsed by the publisher.

## Author’s Disclaimer

The content of this publication does not necessarily reflect the views or policies of the Department of Health and Human Services, nor does mention of trade names, commercial products, or organizations imply endorsement by the U.S. Government.
