# Supplementary material for: Comparative immunogenicity of an mRNA/LNP and a DNA vaccine targeting HIV gag conserved elements in macaques
Source: Front Immunol. 2022 Jul 22;13:945706. doi: 10.3389/fimmu.2022.945706 (PMC9355630; doi:10.3389/fimmu.2022.945706)
Supplement: Supplementary file 1 [file DataSheet_1.pdf]

Supplementary Material

SUPPLEMENTARY FIGURE 1

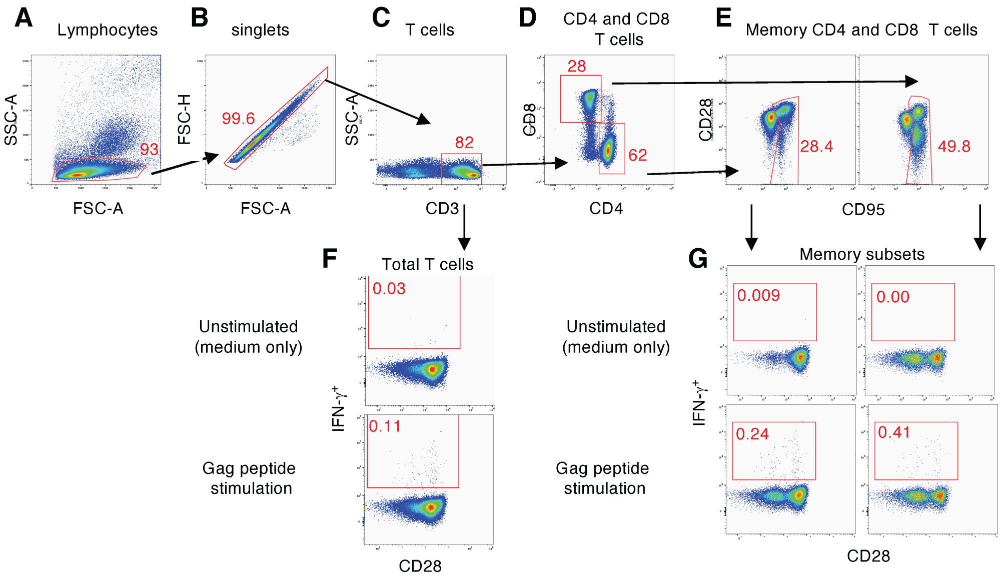

Supplementary Figure 1. Gating strategy for the identification of T cell subsets in peripheral blood of a representative gag mRNA/LNP vaccinated macaque. (A) Lymphocyte gate with side scatter/forward scatter. (B) Single cell gate excluding doublets. (C) $CD3^+$ gate for T cells. (D) $CD4^+$ and $CD8^+$ gates. (E) $CD4^+$ and $CD8^+$ memory (CD28, CD95) T cell subsets. (F) Dot plots showing medium-only stimulation (upper panel) and Gag-specific (lower panel) IFN-γ^+ cells within the total $CD3^+$ population. (G) Dot plots showing medium-only stimulation (upper panel) and Gag-specific (lower panel) IFN-γ^+cells within $CD4^+$ and $CD8^+$ T cell memory subsets.

SUPPLEMENTARY FIGURE 2

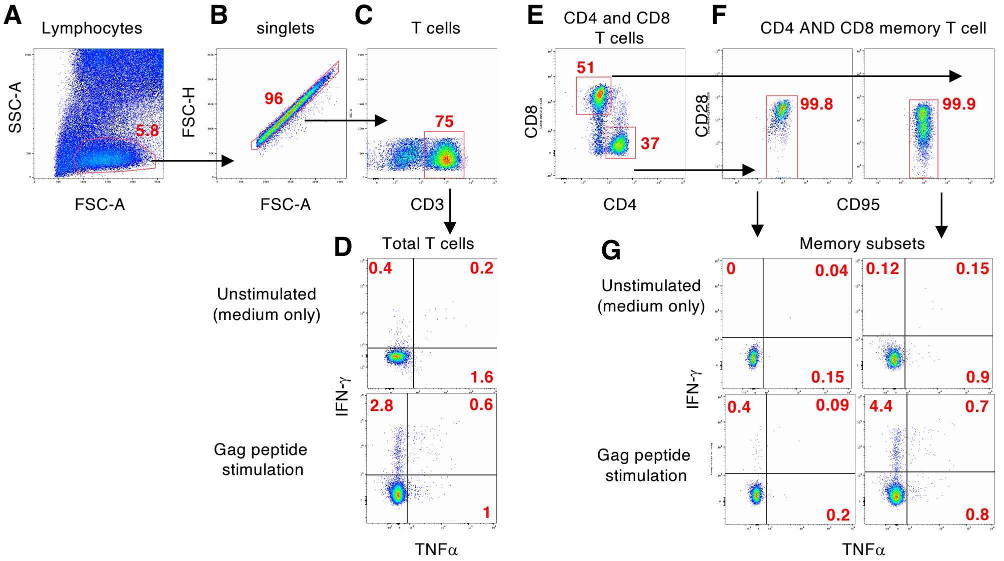

H

Animal ID	Analysis at 2 weeks after last mRNA/LNP vaccination	Dose mRNA/ LNP (μg)	IFN-γ and TNFα producing memory T cells in BAL: CE-specific CD4+ T cells	CE-specific CD8+ T cells	Gag-specific CD4+ T cells	Gag-specific CD8+ T cells
DGHG	3x CE	25	0	0.1	0	0.1
16C190	3x CE	25	0	0.5	0	0.7
DGTE	3x CE	25	0	0	0.3	0
ZM34	3x CE	25	0.4	0	0.2	0
ZN21	3x CE	25	0	0	0	0
16C102	3x gag	25	0	0.7	0.5	0.4
16C141	3x gag	25	0.1	0.1	0.1	0
DGRK	3x gag	25	0	0	0.4	4.3
HXN	3x gag	25	0.2	0	0.9	0
ZM12	3x gag	25	0.1	0	0.5	0.4
16C275	2x CE->1x CE+gag	25	0	0.2	0.2	0.5
16C286	2x CE->1x CE+gag	25	0.4	0	0.3	0.4
DGRW	2x CE->1x CE+gag	25	0	0	0	0
ZM07	2x CE->1x CE+gag	25	0	0	0	0
ZN09	2x CE->1x CE+gag	25	0	2.5	0	0
LL25	2x gag	100	ND	ND	0.2	1.0
LM45	2x gag	100	ND	ND	0.1	0.4
LM88	2x gag	100	ND	ND	0.5	0.5
LR02	2x gag	100	ND	ND	0.3	0.6
LR33	2x gag	100	ND	ND	0.2	0.3

Supplementary Figure 2. Identification of antigen-specific lymphocytes in bronchioalveolar (BAL) fluid from mRNA/LNP vaccinated macaques. (A- H) Gating strategy for the identification of antigen-specific lymphocyte subsets in BAL of a representative *gag* mRNA/LNP vaccinated macaque. (A) BAL with side scatter/forward scatter. (B) Singlets. (C) CD3 gate for T lymphocytes. (D) Dot plots showing medium-only stimulation (upper panel) and Gag-specific (lower panel) IFN-γ^+ cells within the total $CD3^+$ population. (E) CD4 and CD8 gates for $CD3^+$ lymphocytes. (F) $CD4^+$ (left panel) and $CD8^+$ (right panel) memory (CD28, CD95) T cells. (G) Dot plots showing medium-only stimulation (upper panels) and Gag-specific (lower panels) IFN-γ^+ and TNFα^+ memory T lymphocytes within the CD4 (left panel) and CD8 (right panel) memory subsets. (H) Table showing the antigen-specific responses in BAL in the low and high dose mRNA/LNP vaccinated macaques at 2 weeks afer the 3rd and the 2nd vaccination, respectively. A sample is considered positive when value is higher than 0.05 and at least 2-fold over the medium-only control

A

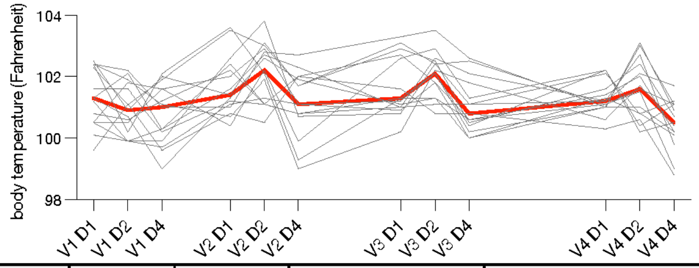

	V1 D2_D1	V2 D2_D1	V3 D2_D1	V4 D2_D1
# of NHP	15	15	15	15
# of NHP with body temp. changes >/=1°F	1	7	3	5
Median body temperature. change [IQR]	1.5 [0]	1.3 [0.7]	1.6 [0.4]	1.5 [1.2]

B

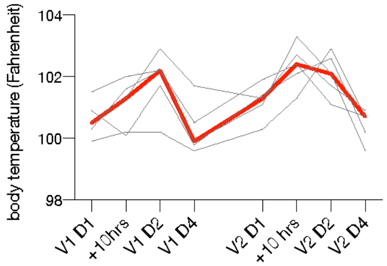

	V1 +10hrs_D1	V1 D2_D1	V2 +10hrs_D1	V2 D2_D1
# of NHP	5	5	5	5
# of NHP with body temp. changes >/=1°F	1	2	3	3
Median body temperature change [IQR]	1.3 [0]	2.2 [0.5]	1.4 [1.2]	1.2 [1.6]

Supplementary Figure 3. Changes in body temperature upon mRNA/LNP vaccinations. Body temperatures (in Fahrenheit) were measured in macaques on day 1, 2, 4 after each mRNA/LNP vaccination. The mRNA/LNP vaccines were administered as (**A**) low (n =15) and (**B**) high dose (n=5) and the data were plotted overtime. The individual animals (grey lines) and median (red lines) were shown. The tables list the number of animals with at least 1° F increase and the median temperature change with [IQR].

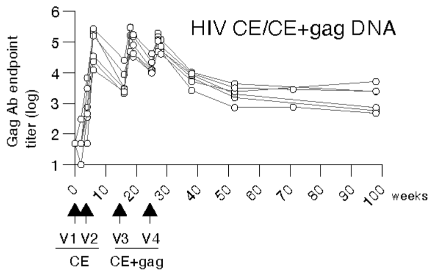

Supplementary Figure 4. HIV CE/CE+gag DNA vaccination of rhesus macaques. Macaques were vaccinated with CE/CE+Gag DNA following the same schedule used for the mRNA/LNP vaccination (Figures 1, group 3). The DNA vaccine (dose: 2 mg prime, 2+2 mg boost) contained IL-12 DNA as vaccine adjuvant and was administered by IM injection followed by electroporation. Plot shows vaccine-induced Gag Ab measured over time as reciprocal endpoint titers (log). The last time points of blood collection were week 95 and 101, respectively, for 3 animals each and these time points were combined plotted as week 98.

SUPPLEMENTARY FIGURE 5

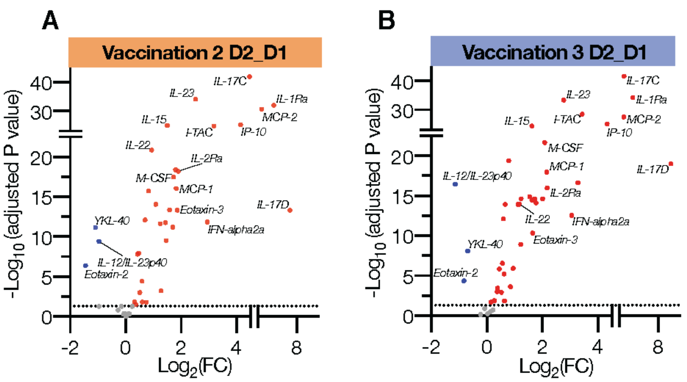

Supplementary Figure 5. Differential expression analysis comparing changes after the 2nd and 3rd vaccination. Mean log2 fold changes (Log2FC) of cytokine levels are shown comparing levels at day 2 to day 1 for all the 15 animals receiving the mRNA/LNP vaccine. Volcano plots of data shown in Figure 3D depict differentially expressed analytes upon the vaccination 2 (**A**) and vaccination 3 (**B**) at day 2 in comparison to day 1. Red dots indicate significant upregulation; blue dots indicate significant downregulation (adjusted p value <0.05 represented by the broken horizontal line).

Supplementary Table 1. Animal description and vaccination details

Animal	Vaccine	Age	Sex	Dose mRNA/ LNP (μg)	Dose gag DNA (mg)	Dose IL-12 DNA (mg)	Dose mRNA /LNP (μg)	Dose gag DNA (mg)	Reported
DGHG	CE RNA	5	M	25					Fig.1
16C190	CE RNA	4	F	25					Fig.1
DGTE	CE RNA	4	M	25					Fig.1
ZM34	CE RNA	6	F	25					Fig.1
ZN21	CE RNA	5	F	25					Fig.1
16C102	gag RNA -> gag DNA	4	F	25				2	Fig.1->5
16C141	gag RNA -> gag DNA	4	F	25				2	Fig.1->5
DGRK	gag RNA -> gag DNA	4	M	25				2	Fig.1->5
HXN	gag RNA -> gag DNA	5	F	25				2	Fig.1->5
ZM12	gag RNA -> gag DNA	6	F	25				2	Fig.1->5
16C275	CE RNA->CE+gag RNA	4	F	25					Fig.1
16C286	CE RNA->CE+gag RNA	4	F	25					Fig.1
DGRW	CE RNA->CE+gag RNA	5	M	25					Fig.1
ZM07	CE RNA->CE+gag RNA	6	F	25					Fig.1
ZN09	CE RNA->CE+gag RNA	5	F	25					Fig.1
LL25	gag RNA	4	M	100					Fig.2
LM45	gag RNA	4	M	100					Fig.2
LM88	gag RNA	4	M	100					Fig.2
LR02	gag RNA	4	M	100					Fig.2
LR33	gag RNA	4	M	100					Fig.2
LI19	gag DNA -> gag RNA	6	M		2		25		Fig. 5->6
LR44	gag DNA -> gag RNA	4	M		2		25		Fig. 5->6
LT10	gag DNA -> gag RNA	4	M		2		25		Fig. 5->6
LT11	gag DNA -> gag RNA	4	M		2		25		Fig. 5->6
LT16	gag DNA -> gag RNA	4	M		2		25		Fig. 5->6
5698[a]	CE->CE+gag DNA	5	M		2->2+2[b]	0.2			Fig.1
5699[a]	CE->CE+gag DNA	5	M		2->2+2[b]	0.2			Fig.1
5700[a]	CE->CE+gag DNA	6	M		2->2+2[b]	0.2			Fig.1
570[a]	CE->CE+gag DNA	6	M		2->2+2[b]	0.2			Fig.1
5702[a]	CE->CE+gag DNA	6	M		2->2+2[b]	0.2			Fig.1
5703[a]	CE->CE+gag DNA	6	M		2->2+2[b]	0.2			Fig.1
L982[c]	gag DNA	13	M		1	0.2			Fig.2
P574[c]	gag DNA	6	F		1	0.2			Fig.2
R067[c]	gag DNA	3	M		1	0.2			Fig.2
R288[c]	gag DNA	3	M		1	0.2			Fig.2
ZM01	gag DNA -> gag RNA	3	M		2	0.2			Fig.6
ZM04	gag DNA -> gag RNA	3	F		2	0.2			Fig.6
ZM06	gag DNA -> gag RNA	3	M		2	0.2			Fig.6

[a]Hu X, et al. J Immunol: 197:3999-4013 (2016)
[b]2 mg CE DNA prime ->2 mg CE DNA+2mg gag DNA boost
[c]Kulkarni V, et al. PLoS One:9 e111085 (2014).

Supplementary Table 2. Cytokines and Chemokines (N=61) tested

Analytes with changes (N=35)	Eotaxin	Analytes with minimal or no change (N=9)	IL-16
	Eotaxin-2		IL-1α
	Eotaxin-3		IL-2
	Fractalkine		MDC
	GRO-a		SDF-1a
	I-TAC		CTACK
	IFN-α2a		ENA-78
	IL-12/IL-23p40		MIP-3α[c]
	IL-15		FLT3L[c]
	IL-17A/F	Analytes below detection threshold (N=17)	G-CSF
	IL-17B		GM-CSF
	IL-17C		I-309
	IL-17D		IFN-γ
	IL-17F		IL-10
	IL-18		IL-12p70
	IL-1Ra		IL-13
	IL-22[a]		IL-17A
	IL-23		IL-1β
	IL-2Ra[b]		IL-4
	IL-6		IL-5
	IL-7		IL-8
	IL-9*		MCP-3
	IP-10		MIP-5
	M-CSF		TARC
	MCP-1		TNF-α
	MCP-2		TNF-β
	MCP-4		
	MIF		
	MIP-1α		
	MIP-1β		
	MIP-3β		
	TPO		
	TRAIL		
	VEGF-A		
	YKL-40		

[a]Analyte below threshold of detection in high-dose vaccine
[b]Analyte absent from MSD kit used in high dose analysis
[c]Analyte affected in high-dose mRNA/LNP vaccine group only
